# The Lebanese COVID-19 Cohort; A Challenge for the ABO Blood Group System

**DOI:** 10.3389/fmed.2020.585341

**Published:** 2020-11-20

**Authors:** Athar Khalil, Rita Feghali, Mahmoud Hassoun

**Affiliations:** ^1^Department of Pulmonary and Intensive Care Unit, Rafik Hariri University Hospital, Beirut, Lebanon; ^2^Department of Laboratory Medicine, Rafik Hariri University Hospital, Beirut, Lebanon

**Keywords:** COVID-19, ABO, SARS–CoV-2, infection predisposition, MENA (Middle East and North Africa)

## Abstract

A sudden outbreak of pneumonia caused by the Severe Acute Respiratory Syndrome Coronavirus 2 (SARS-CoV-2) has rapidly spread all over the world facilitating the declaration of the resultant disease as a pandemic on March 2020. Predisposing factors for acquiring COVID-19 and for developing a severe form of this disease were postulated to be related to the epidemiological, clinical, and genetic characteristics of the patients. Biological markers such as the ABO blood group system were amongst these factors that were proposed to be linked to the variability in the disease course and/or the prevalence of the infection among different groups. Herein, we conducted the first retrospective case-control study from the Middle East and North Africa that tackles the association between the blood group types and the susceptibility to, as well as the severity of, SARS-CoV-2 infection. Contrary to the most acknowledged hypothesis, our results challenged the significance of this association and questioned the role of the ABO blood group system in dictating the severity of this disease. For future similar studies, we endorsed analyzing larger cohorts among different populations and we encouraged implementing more rigorous approaches to diminish the potential confounding effect of some underlying comorbidities and genetic variants that are known to be associated with the ABO blood group system.

## Introduction

In December 2019, a sudden outbreak of pneumonia has emerged in Wu Han City, China and has spread all over the world (1). This coronavirus disease 2019 (COVID-19) as named by the World Health Organization (WHO), is caused by one of the Beta-coronaviruses that primarily targets the human's lower respiratory tract. COVID-19 is mainly characterized by dry cough, dyspnea, fever, and bilateral lung infiltrates upon imaging. The primary cause of death among the infected patients remains to be the acquired severe respiratory failure (2). The fast spread of this contagious disease allowed it to reach around 200 countries in a short period of time and has facilitated the declaration of COVID-19 outbreak as a pandemic on the 11th of March 2020 (3). Up till this date, the global number of confirmed positive cases has exceeded 10 million and caused more than 500,000 deaths (4). In Lebanon, the official authorities had forced strict measures shortly after detecting the first case of COVID-19. This fast action of declaring a state of emergency in the country was among the factors that helped in an initial successful containment of the disease. So far, the Ministry of Public Health in Lebanon has confirmed 1885 cases of COVID-19 and 36 associated deaths (5, 6).

**Figure 1:**
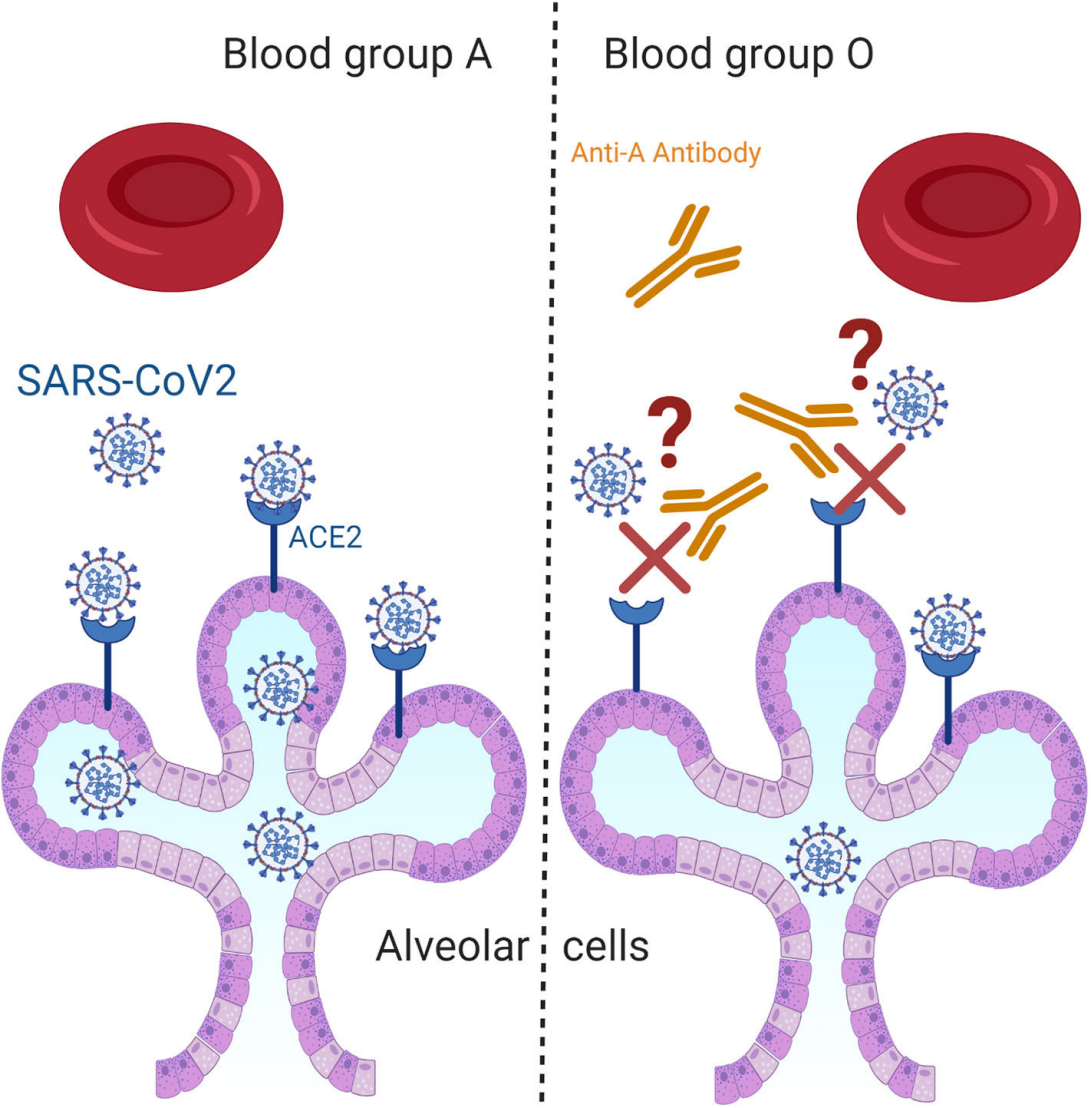
Questioning the hypothetical biological mechanism that associates ABO blood groups with COVID-19. Among the most proposed hypothetical biological mechanisms that could explain the increased risk for SARS-CoV-2 infection among blood group A individuals as compared to that of blood group O is the presence of anti-A antibodies in the serum of O blood type carriers that can inhibit the virus–cell adhesion process.

The predisposition for acquiring COVID-19 and for developing a severe form of this disease is known to be affected by some epidemiological and clinical characteristics of a population. The main studied risk factors are age, sex, and some chronic conditions such as diabetes and cardiovascular diseases (7, 8). Yet, we are still lacking biological markers that can predict the prevalence of this infection or that can explain the variability in the disease course among different groups (9). From that perspective and due to the highly tackled association between blood groups and the susceptibility/severity of various diseases such as SARS-CoV-1, *P. falciparum, H. pylori*, Norwalk virus, hepatitis B virus, and *N. gonorrhoeae*, investigations on their association with COVID-19 cases was evaluated (10). Zhao et al. were the first to report an association between ABO blood groups and the susceptibility to SARS-CoV-2 (11). Afterwards, several studies has emerged to pinpoint again on the increased risk for infection and mortality by SARS-CoV-2 among blood group A individuals as compared to that of blood group O (9–12). The explanation of this linkage was attributed to several hypotheses such as the presence of an extra sugar N-acetyl galactosamine on the surface of blood group A cells that can possibly propose more pathogen-host contact or the presence of anti-A antibodies IgGs in the serum of O blood type carriers that can inhibit the virus–cell adhesion process (Figure 1) (12, 13). Recently, a newly published article in The New England Journal of Medicine presented a genetic piece of evidence that established a potential role of the ABO blood-group system as a risk factor for acquiring COVID-19. In their article, Severe Covid-19 GWAS Group et al. have associated the rs657152 variant that coincided on the ABO blood group locus (9q34.2) with COVID-19–induced respiratory failure (14).

Knowing that all the above mentioned articles provided a consistent association between the ABO blood system and COVID-19 disease, yet, all of these studies emphasized on the importance of investigating this association among larger clinical datasets from different populations. For this reason, we decided to evaluate the association the ABO blood group system and the susceptibility as well as the severity of SARS-CoV-2 infection in our Lebanese Cohort.

## Methods

### Data Source

Medical records for COVID-19 patients and their clinical characteristics were obtained from the electronic medical database at Rafik Hariri University Hospital (RHUH). The retrieved variables for COVID-19 patients were age, sex, blood group, and severity of their disease. For the control cases, only blood group types were retrieved.

### Study Design

This is a retrospective case-control study with 146 patients diagnosed with COVID-19 and 6,497 individuals in the control group. The case group consisted of COVID-19 patients admitted to RHUH between February 2020 and June 2020. The diagnosis of COVID-19 was confirmed by a positive real-time reverse transcriptase polymerase-chain-reaction test for SARS-CoV-2 on nasal and pharyngeal swab specimens from patients. For the control group, blood groups data was retrieved for all individuals who were admitted to RHUH between 01/01/2018 till 24/06/2020. For the control group, all blood samples were typed by forward test tube method while for the case group the typing was done by both forward and reverse testing.

Severity classification criteria was adopted from the interim guidelines of the World Health Organization and the National Health Commission of China. The disease severity of COVID-19 patients was categorized into four types: mild, moderate, severe, and critical (15, 16). Given the relatively small sample size of our cohort and the low number of severe and critical cases, we combined these two categories together. Briefly, (1) Mild cases are patients diagnosed with upper respiratory symptoms without imaging abnormalities (2) Moderate patients are those who presented with at least two of the following symptoms: dyspnea, cough, or body temperature > 38°C and had imaging abnormalities, (3) Severe cases are those characterized by respiratory distress with respiratory rate ≥ 30 Breaths Per Minute (BPM), O2 Saturation ≤ 93%, and PaO2/FiO2 ≤ 300 mmHg, while (4) critical cases are those who suffered from acute respiratory failure necessitating non-invasive or/and invasive ventilation and developed multi organ failure such as renal, failure or heart failure and shock (15).

This study was approved by the Ethical Committee of RHUH. Due to the retrospective nature of this study and since no identifying information related to participants was included, written informed consent was waived.

### Statistical Analysis

Statistical computations were performed using IBM SPSS statistics 24. Descriptive statistics were conducted to determine the characteristics of the overall cohort. Frequencies and percentages were reported for categorical variables while means and SD were reported for the continuous ones. Data were analyzed by Chi-square (χ^2^) test and Fisher's exact test when applicable. We compared each blood group against all others using a 1 x 2 contingency table to determine effect sizes for each blood group in our COVID-19 cohort. The outcome variable was categorized as following: mild, moderate or severe/critical. Binary logistic regression analysis adjusted for age and gender was done for each blood group vs. the rest of the blood groups. Results were reported as odds ratio with a 95% confidence interval. P-values of less than or equal to 0.05 were considered statistically significant.

## Results

### The ABO Blood Group Distribution Among COVID-19 Patients and the Control Group

**Figure 2:**
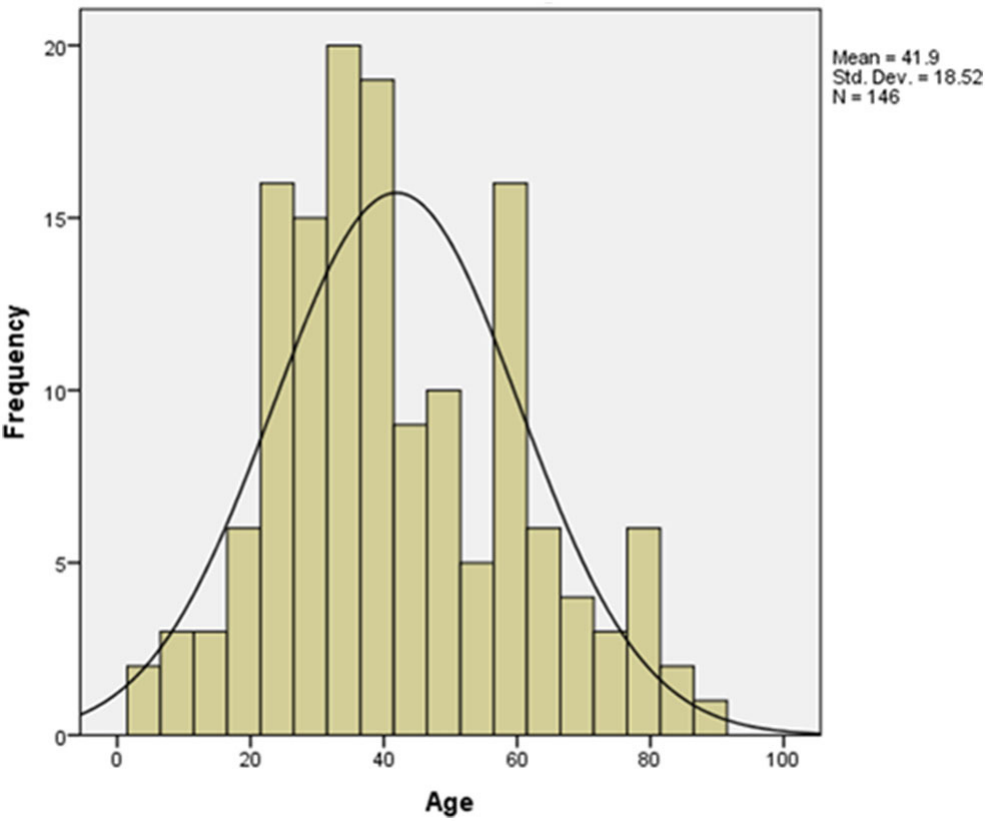
Age distribution pattern among COVID-19 patients. The mean age for the 146 involved patients in this study was 41.9 (*t*-test = 27.336, *p* value < 0.001).

A total of 146 patients diagnosed with COVID-19 were enrolled in this study. The mean age of the case group was 41.9 years old (interquartile range (IQR): 28–57 years, SD = 18.52). Patients who aged between 22 and 40 years old constituted the largest portion of our cohort (45.2%) (Figure 2, Table 1). The frequency of males (*n* = 97, 66.4%) was higher than that of the females (*n* = 49, 33.6%) (Table 1). Frequencies of blood groups showed that the highest percentage belong to blood group A (40.4%) followed by blood group O (35.6%) and then blood group B (17.1%) and AB (6.8%) (Table 1). Mild cases scored the highest percentage in this cohort (*n* = 72, 58.6%) while those with severe or critical symptoms presented only 13.7% of the cases (Table 1).

**Table 1:** Clinical characteristics of COVID-19 patients including sex, ABO blood groups, and severity outcome of SARS-CoV2 infection.

	**Variables**	**Numbers (Total = 146)**	**Percentages (Total = 100%)**
Gender	Male	97	66.4%
	Female	49	33.6%
Severity of COVID-19	Mild	72	49.3%
	Moderate	54	37%
	Severe/ Critical	20	13.7%
ABO blood group	A	59	40.4%
	AB	10	6.8%
	B	25	17.1%
	O	52	35.6%

Our control group constituted a total of 6479 individuals who were admitted to the RHUH between 1/1/2018 and 1/6/2020. Blood group O was the highest detected blood group with 2,573 cases (39.7%) while AB blood group scored the lowest number with 352 cases (5.4%). Although the percentage of patients with blood group A was higher among COVID-19 cases as compared to that of the control group (40.4% vs. 35.8%, *P* = 0.25), while that of blood group O was lower (35.6% vs. 39.7%, *P* = 0.32), these differences did not reach statistical significance (Table 2).

**Table 2:** The ABO blood group distribution among COVID-19 patients and normal controls.

**Blood groups**	**COVID-19 patients *N* = 146 (100%)**	**Control group *N* = 6,479 (100%)**	**χ^2^**	***P*-value**
A	59 (40.4%)	2,322 (35.8%)	1.30	0.25
B	25 (17.1%)	1,232 (19%)	0.33	0.56
O	52 (35.6%)	2,573 (39.7%)	1.00	0.32
AB	10 (6.9%)	352 (5.4%)	0.55	0.46

### The Effect of Age, Sex, and Blood Group on Dictating the Severity of COVID-19

**Figure 3:**
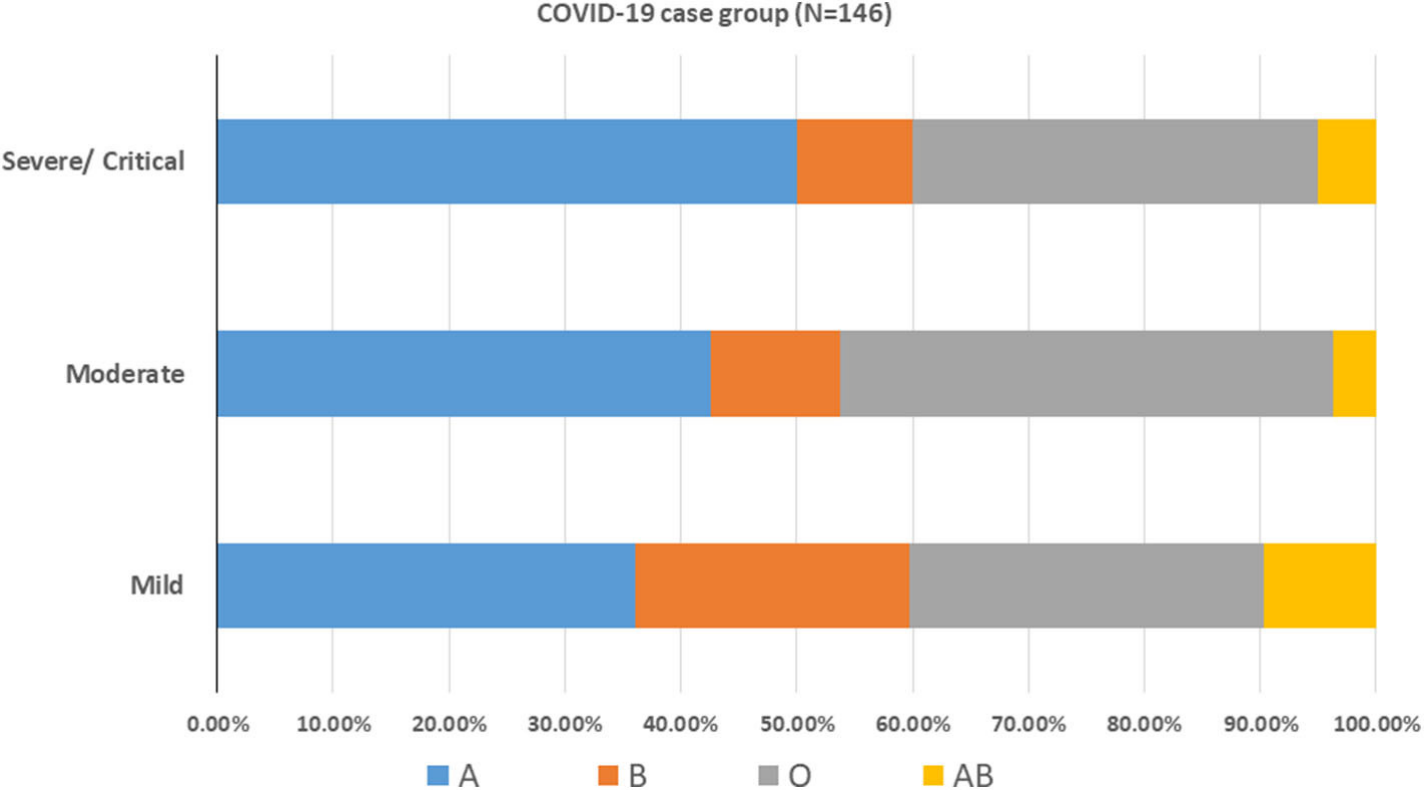
ABO blood group distribution pattern among COVID-19 patients. COVID-19 cases were stratified according to the disease severity into mild, moderate and severe/critical cases.

After stratifying our data based on the severity outcome of the disease, only the age factor showed a significant association with the three categories of severity (*p* < 0.001). Mild cases were mostly patients below 40 years old, moderate cases were those between 40 and 59 years old (46.3%) while severe and critical cases belonged mainly to patients aging above 60 years old (55%) (Table 3). The A blood group was predominant among mild and severe/critical cases while the proportion pf patients with either blood group A or blood group O was the same for moderate cases (Figure 3). Yet, neither the blood group system nor the gender reached a statistical significance in association with the severity of the disease. This is reasonable since in all the outcome categories, blood group A and the male gender were predominant (Table 3). Taking into consideration that age as an independent variable had a significant association with COVID-19 outcome and since previous studies have revealed a potential effect of gender on the severity of the disease, we went further in our analysis by adjusting for these factors. Thus, we assessed whether any of the blood groups could be associated with the mild or the severe/critical form of the disease while adjusting for the age and gender variables. None of the blood groups showed to be a significant risk factor for developing a severe form of the disease. Only individuals with blood group AB had higher odds for the mild form of the disease (OR = 4.613, <0.108) with a borderline significance (Table 4).

**Table 3:** The unadjusted association between the stratified outcome of COVID-19 and various variables including age, sex, and ABO blood groups.

		**Severity of the disease**			**χ^2^**	***P*-value**
		**Mild**	**Moderate**	**Severe/Critical**		
Blood group	A	36.1%	42.6%	50%	1.464	0.493
	B	23.6%	11.1%	10%	3.844	0.149
	O	30.6%	42.6%	35%	1.966	0.386
	AB	9.7%	3.7%	5%	1.638	0.446
Total	100%	100%	100%			
Sex	Female	34.7%	35.2%	25%	0.714	0.717
	Male	65.3%	64.8%	75%		
Total	100%	100%	100%			
Age group	≤ 21	19.4%	0%	0%	16.146	0.000
	22-40	65.3%	29.6%	15%	24.681	0.000
	41-59	12.5%	46.3%	30%	17.935	0.000
	≥60	2.8%	24.1%	55%	3.544	0.000
Total	100%	100%	100%			

**Table 4:** Logistic regression analysis adjusted by age and sex for assessing the effect of each blood group on COVID-19 outcome.

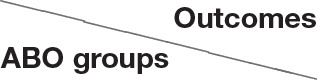	**Mild. vs. not mild**	**Severe. vs. not severe**
A—Non A	26 (44.1%)	10 (16.9%)
	OR = 1.029 CI: 0.414-2.559 *P* = 0.951	OR = 0.752 CI: 0.238-2.378 P = 0.628
B—Non B	17 (68%)	2 (8%)
	OR = 2.847 CI: 0.905-9.959 *P* = 0.73	OR = 0.879 CI: 0.170-4.538 *P* = 0.877
O—Non O	22 (42.3%)	7 (13.5 %)
	OR = 0.340 CI: 0.135-0.855 *P* = 0.22	OR = 1.536 CI: 0.487-4.849 *P* = 0.464
AB—Non AB	7 (70%)	1 (10%)
	OR = 4.613 CI: 0.717-29.684 *P* = 0.108	OR = 0.784 CI: 0.083-7.393 *P* = 0.832

*OR, Odds Ratio; CI, Confidence interval; P, P value*.

## Discussion

This retrospective study was done at the Rafik Hariri University Hospital (RHUH)– formerly known as Beirut Governmental University Hospital. Currently, RHUH is the leading center for COVID-19 testing and treatment in the country. Herein, we analyzed the distribution of the ABO blood groups among the COVID-19 cohort of the Lebanese citizens while taking into consideration that of the control group (*n* = 6497). For our knowledge, this is the first study in the MENA region that tackles the association between the ABO blood groups and the susceptibility to COVID-19 infection while accounting for the population blood group distribution pattern. Globally, we are the first to question the significance of the association of the ABO blood system as a risk factor for the stratified outcomes of COVID-19.

Primarily, in this study we reassured the previously established pattern of the ABO blood groups distribution in our population by showing that blood group O is the predominant one (17). Yet, blood group A was the most prevalent blood group among our COVID-19 cohort (40.4%, *P* < 0.001) which is consistent with previous reports (18). This association lost it significance when the distribution of the blood groups among the control cases was taken into consideration. Thus, since the distribution of blood groups among our COVID-19 patients showed a comparable scattering pattern with that of the general population, the significance of the association between blood groups and the susceptibility to the infection was not distinguished (19). In this regard, it should be well-noted that some of the previously conveyed data did not account for the distribution of blood groups among their general population while those who took into consideration this calculation involved low representing numbers that might not be sufficient for a significant reflection to the entire population (9, 18, 20). Moreover, discrepancies between the conveyed results prevented a firm agreement on a specific blood group to be a risk factor for acquiring SARS-CoV-2. Although initially blood group A was proposed as a the risk factor for contracting COVID-19, yet, subsequent studies from Iran, Saudi and Shenzhen has proposed that AB individuals are rather at higher risk to acquire this disease as compared to other blood groups (18, 21). This inconsistent association could be explained by the different epidemiological patterns among various ethnicities which pinpoint on the importance of including larger cohorts from different populations for more precise associations and conclusions.

So far, multiple risk factors for developing a severe form of SARS-CoV-2 infection are established. Our data re-assured the significance of age in enhancing the severity of the infection among patients. The age factor is confirmed to be associated with poor outcomes characterized by severe symptoms with non-improvement and higher mortality rates (16, 22). This was explained by the fact that the strong host innate immune responses among older individuals can cause an insufficient control of viral replication and a prolonged pro-inflammatory responses which can in turn lead to a marked decline in cell-mediated and humoral immune function (16). For gender distribution, the higher infection incidence among men in our cohort was similar to the globally accepted pattern. But when stratified by disease severity, the male gender was not considered as a significant risk factor for developing a severe form of the disease which is consistent with the previously published results (23).

Since ABO blood groups and/or other comorbidities such as cardiovascular diseases were speculated to be prognostic markers for COVID-19 and not risk factors that predispose for SARS-CoV-2 infection, we studied the association of this system with the stratified severity outcomes of the disease. (24) Herein, we were unable to report a significant association between any of the blood groups and the severity outcome among COVID-19 patients even when other influencing factors were taken into consideration. Data from several other cohorts have also revealed a non-significant association between the ABO system and the severity of infection, intensive care unit (ICU) admission, intubation and death among patients (9, 10, 21). Additionally, the recent GWAS results revealed a non-significant difference in the blood-group distribution between patients receiving only supplemental oxygen and those receiving mechanical ventilation of any kind (14). Thus, our results could be added to the list of data that questioned the significance of presenting the blood group system as a risk factor for dictating COVID-19 outcome. One suggested explanation is that once SARS-CoV2 infection is fully established, it can replicate in the individual's epithelial cells by exhibiting the individual's antigen, and thus rendering the individual's ABO antibodies ineffective against the newly produced viruses.

The flood of fast-paced research and reporting milieu concerning this pandemic, especially when dealing with preprint servers, is causing confusion and is triggering controversial information. Major pitfalls in COVID-19 research concerning the involvement of the blood group system were identified. The analysis of the data in most of the published studies have neglected the effect of other risk factors and comorbidities that are known to worsen SARS-CoV-2 infections and are distributed differently among blood groups. For example, blood group A is considered a significant risk factor for coronary heart disease (CHD), venous deep thromboembolism (VTE), fever, cough, dyspnea, sore throat, chest pain/distress, and fatigue which are all implicated in COVID-19 prognosis (20, 25, 26). A recent study has established a significant association between some COVID-19 commodities/variables and blood group A but these cofounders were not adjusted when studying the independent effect of blood groups on the severity outcome (9). Similarly, allele A lost its significance as a risk factor for susceptibility and mortality in COVID-19 when polymorphisms of angiotensin-converting enzyme 1 (ACE1) and complement component 3 (C3) were added to the multivariate regression model (27). While we do acknowledge that the main limitation of our study is the relatively small sample size, we are the first to evaluate the association between different blood groups with the stratified outcome of COVID-19 while taking into consideration the gender and age factors. In conclusion, larger cohorts with more rigorous approaches are recommended to diminish the potential confounding effects of some underlying comorbidities and genetic variants that are known to be associated with the ABO blood group and can be over-presented in COVID-19 cohorts (25).

## Data Availability Statement

The original contributions presented in the study are included in the article/supplementary materials, further inquiries can be directed to the corresponding author/s.

## Ethics Statement

The studies involving human participants were reviewed and approved by Ethical Committee of RHUH. The ethics committee waived the requirement of written informed consent for participation.

## Author Contributions

AK, RF, and MH contributed to the conception or design of the work. AK contributed to the acquisition, analysis, or interpretation of data for the work. AK drafted the manuscript. AK, RF, and MH critically revised the manuscript. All gave final approval and agree to be accountable for all aspects of work ensuring integrity and accuracy.

## Conflict of Interest

The authors declare that the research was conducted in the absence of any commercial or financial relationships that could be construed as a potential conflict of interest.
